# Supplementary material for: Patterns of Intron Gain and Loss in Fungi
Source: PLoS Biol. 2004 Nov 30;2(12):e422. doi: 10.1371/journal.pbio.0020422 (PMC532390; doi:10.1371/journal.pbio.0020422)
Supplement: Table S1 — Also available at http://genes.mit.edu/NielsenEtAl/. (4.3 MB ZIP). [file pbio.0020422.st001.zip › NielsenEtAl/html/971.html]

AN4299.1.NCU08949.1.MG06000.1.FG10303.1


```
 CLUSTAL W (1.82) Multiple Sequence Alignments - Introns Inserted


Sequence 1: AN4299.1	151 aa
Sequence 2: MG06000.1	150 aa
Sequence 3: FG10303.1	150 aa
Sequence 4: NCU08949.1	171 aa
Alignment Length: 171 aa
Number Identitical Residues: 84 aa
Alignment Score (without introns) 4107


MG06000.1 	-MSLHY1--------------------~LPPVKPSAIALGTVFCQTAELAV1LTPIFGAT
NCU08949.1	MTSISN~SPQSFILLDTGSAQKHLVSD1LPPVKPSAIALGTIFNHATELAI~MTPLFGQT
FG10303.1 	-MSLHY~--------------------~LPPVKPSAIALGTAFNHGVELAV~LAPIFGQT
AN4299.1  	MATLHY1--------------------~LPPVKPSAIAAGTIFTHTASLGI~LAPVFGDT
          	  ::                        ********** ** * : ..*.: ::*:** *

MG06000.1 	YNRAKAANTKEEFLRSKEASSAGIAWGTSLVGSALQSYGVGALINATATLSYKGSAYLGA
NCU08949.1	YQRAKISNSKEEFIKSREATSAVMAWGSSLVGSALQSYGIGALINATGTLSYRGAAYLGG
FG10303.1 	YQRAKSSNTKEEFIRSKEASGAALAWGSSLTGSALQAYGVGALINATGTLTYKGAAYLGA
AN4299.1  	YHRAQAANSKEEFVRSKEAAGAAAAWGSSLVGSAVQTYGVAALINATGTLSYKGAAYLGS
          	*:**: :*:****::*:**:.*  ***:**.***:*:**:.******.**:*:*:****.

MG06000.1 	LIFMATSAPG0YISQVFLEKRPLETVGVSVVAKVMETVGLACVLTWWGTRTNPFE
NCU08949.1	LIFAATSAPG~FISEFFVEKRAFETVGINVLAKLFETVGLSVFLTWWGTRTNPFE
FG10303.1 	LIFFATSAPG0YVSQIFVEKRPLDTVGVSLATKLVETLGLSVFLTWWGTRTNPFE
AN4299.1  	LIFFASSAPS0FVSQIFTEKRPLDTVAVGAVARVFETVGLSLFLTWWGTRTNPFD
          	*** *:***. ::*:.* ***.::**.:.  :::.**:**: .***********:
```
